# Transition clinic for neurodevelopmental disorders: preliminary findings from an interdisciplinary model for adolescence to adulthood

**DOI:** 10.3389/fpsyt.2026.1882420

**Published:** 2026-07-22

**Authors:** Hasan Ali Güler, Mesut Güngör, Fatih Mehmet Akif Özdemir, Mehmed Ediz Çelik, Ülkü Hüma Akbulut, Esra Nakipoğlu, Ali Kandeğer

**Affiliations:** 1 Department of Child and Adolescent Psychiatry, Selçuk University Faculty of Medicine, Konya, Türkiye; 2 Department of Child Neurology, University of Health Sciences Ankara Bilkent City Hospital, Ankara, Türkiye; 3 Department of Child Neurology, Selçuk University Faculty of Medicine, Konya, Türkiye; 4 Department of Psychiatry, Selçuk University Faculty of Medicine, Konya, Türkiye

**Keywords:** ADHD, autism spectrum disorder, genetic, neurodevelopmental disorders, neurology, transition clinic

## Abstract

**Introduction:**

Neurodevelopmental disorders (NDDs) are childhood-onset conditions that often persist into adulthood and are associated with substantial functional impairment and complex psychiatric comorbidities. However, the transition from child and adolescent to adult mental health services often remains fragmented, contributing to delayed recognition and unmet clinical needs. This study aimed to describe an interdisciplinary transition clinic model for individuals with NDDs and to present preliminary findings regarding diagnostic outcomes and clinical characteristics.

**Methods:**

This study was conducted in a tertiary-care transition clinic jointly operated by consultant child and adolescent psychiatrists and adult psychiatrists, with psychiatry residents participating as observers. All patients were also evaluated by a consultant neurologist as part of the interdisciplinary assessment process. The clinic followed a three-stage model consisting of referral and pre-assessment, interdisciplinary diagnostic evaluation, and integrated treatment and follow-up. Adolescents and young adults aged 15–21 years with suspected or established NDDs underwent psychiatric, neurological, and when clinically indicated, genetic evaluations. Descriptive analyses were performed for diagnostic distributions, newly identified disorders, and psychotropic medication use.

**Results:**

A total of 68 participants were included (mean age = 17.90 ± 1.90 years; 54.4% male). The median number of diagnoses increased from 1 (range: 0–4) before the transition clinic assessment to 3 (range: 1–11) after evaluation. Attention-deficit/hyperactivity disorder was the most frequent NDD (66.2%), while 80% of autism spectrum disorder diagnoses were identified during the transition clinic process. Social (pragmatic) communication disorder, social anxiety disorder, avoidant and borderline personality disorder, and body-focused repetitive behavior disorders were frequently newly identified during transition clinic assessments. Structural MRI findings were observed in 26.7% of scanned participants.

**Discussion:**

These findings suggest that interdisciplinary transition clinics may facilitate the identification of previously unrecognized NDDs and psychiatric diagnoses during adolescence and emerging adulthood. Integrated psychiatric, neurological, and genetic assessment models may also contribute to continuity of care and psychiatric resident education.

## 1 Introduction

Neurodevelopmental disorders (NDDs) are conditions with onset in childhood that frequently persist into adulthood and are associated with significant functional impairment (1). In particular, attention-deficit/hyperactivity disorder (ADHD) and autism spectrum disorder (ASD) often demonstrate a persistent course across the lifespan, contributing to substantial academic, occupational, and social difficulties in adult life (2).

In addition to core diagnostic symptoms, individuals with NDDs frequently exhibit impairments in executive function which play important roles in social cognition, behavioral adaptation, and communication (3). Despite the high clinical burden associated with NDDs, mental health services for complex NDD presentations frequently remain fragmented, contributing to delays in diagnosis and access to appropriate support (4).

The concept of transition encompasses two parallel processes: the developmental transition of the individual into adulthood and the transfer of care from child to adult health services. When transition is approached merely as an administrative transfer, developmental and psychosocial needs may remain insufficiently addressed, thereby compromising its holistic nature (5). Individuals with NDDs are among the most vulnerable groups during this period, with an increased risk of discontinuity of care and unmet clinical needs (6). Accordingly, recent European guidelines emphasize that effective transition requires structured, interdisciplinary planning and active involvement of both young people and their caregivers (7).

In addition to psychiatric complexity, individuals with NDDs frequently present with a substantial burden of medical comorbidities and increased healthcare utilization, reflecting the overall clinical complexity of this population (8). Notably, transition-aged individuals with NDDs exhibit high rates of neurological and genetic comorbidities, which tend to become more pronounced in late adolescence and early adulthood (9). These findings suggest that, beyond mental health care, systematic evaluation of neurological and other medical comorbidities may be an important component of transition planning.

Another important barrier in the transition process is the limited expertise of mental health professionals in managing NDDs. Clinicians frequently report limitations in education and training related to NDDs (10). In this context, interdisciplinary transition clinics may offer a valuable model by providing comprehensive biopsychosocial assessment while facilitating integration across multidisciplinary clinical and research teams.

The aim of the present study was to describe a transition clinic for individuals with NDDs, involving collaboration between child and adolescent psychiatry, adult psychiatry, and neurology, with genetic consultation when indicated. We present preliminary findings from this model, changes in diagnostic profiles before and after transition clinic assessment, rates of newly identified psychiatric disorders, findings from neurological and genetic evaluations, and patterns of medical treatment. To our knowledge, a structured clinical model integrating psychiatric and neurological evaluation within a dedicated transition setting for NDDs has not been previously described.

## 2 Materials and methods

### 2.1 Study design and outcomes

This study represents a service evaluation of a specialized interdisciplinary transition clinic for individuals with NDDs. The primary aim was to describe the structure and organization of the clinic and to present preliminary clinical findings obtained during routine service delivery. Primary outcomes included changes in diagnostic profiles following transition clinic assessment, rates of newly identified NDDs and psychiatric diagnoses, neurological and genetic evaluation findings, and the distribution of psychotropic medication use.

### 2.2 Study center and transition clinic framework

This study was conducted collaboratively by the Adult Neurodevelopmental Disorders Clinic, Department of Psychiatry, and the Adolescent Neurodevelopmental Disorders Clinic, Department of Child and Adolescent Psychiatry, at Selçuk University Faculty of Medicine. The study was carried out at Selçuk University Faculty of Medicine Hospital, a tertiary care center in Konya, Türkiye, within a specialized transition clinic designed for individuals with NDDs. The clinic was jointly operated by consultant specialists in child and adolescent psychiatry, adult psychiatry, and neurology. Psychiatry residents also participated in the clinical process as observers.

### 2.3 Aims of the transition clinic

The transition clinic was established to provide comprehensive interdisciplinary assessment and continuity of care for adolescents and young adults with NDDs during the transition from child and adolescent mental health services to adult services. In addition to its clinical role, the clinic was designed to support interdisciplinary collaboration in clinical and scientific practice and to provide opportunities for resident participation in the evaluation of complex NDD and psychiatric presentations.

### 2.4 Ethical approval and study period

The study covered the period between March 2025 and March 2026. Ethical approval was obtained from the Selçuk University Local Ethics Committee (Number: 2025/401). Written informed consent was obtained from all participants and from the parents of participants under 18 years of age. The study was conducted in accordance with the principles of the Declaration of Helsinki.

### 2.5 Procedure and structure of the transition clinic

**Figure 1:**
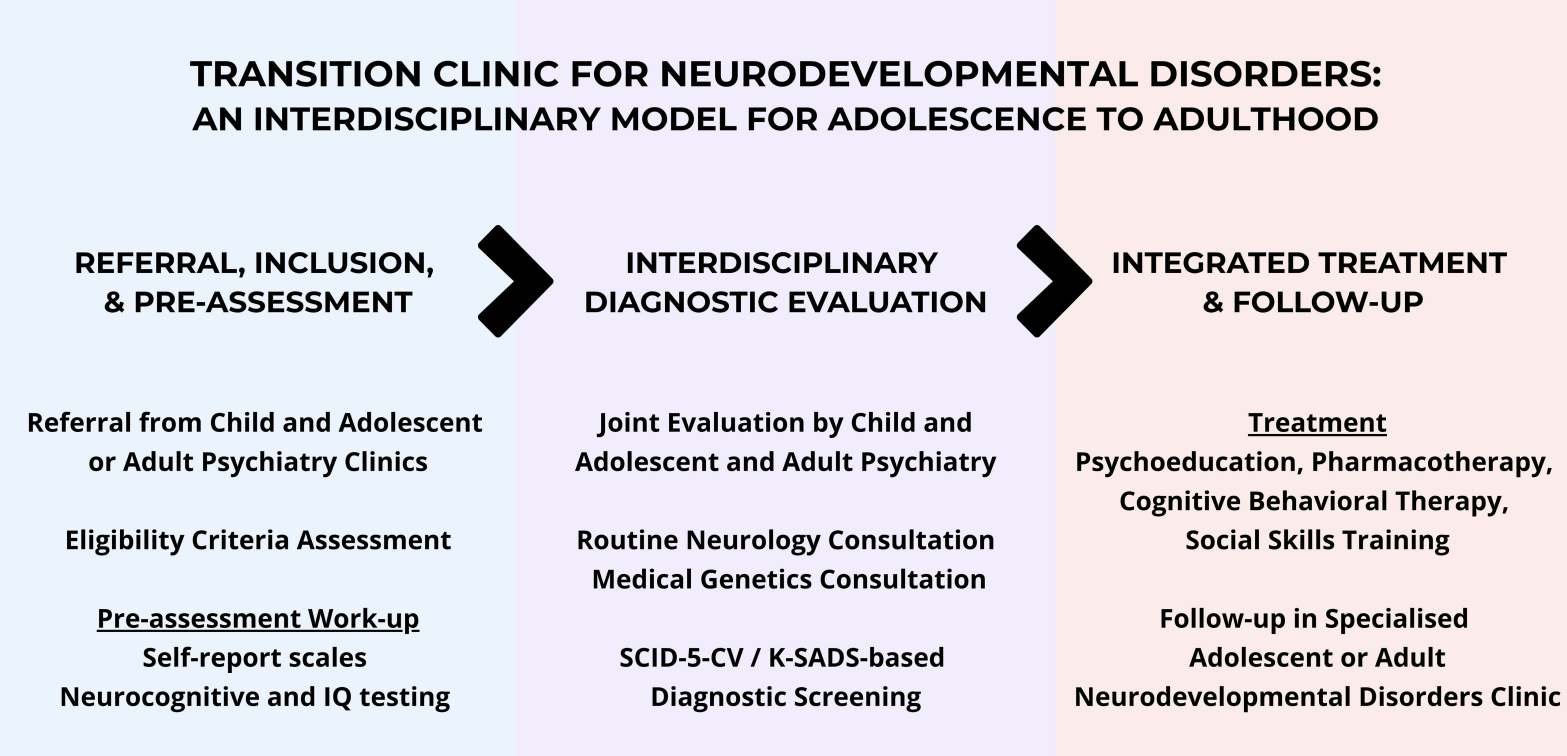
Workflow of the Transition Clinic for Neurodevelopmental Disorders, illustrating the three phases: referral, inclusion, and pre-assessment; interdisciplinary diagnostic evaluation; and integrated treatment and follow-up.

The workflow and organizational structure of the transition clinic are presented in **Figure 1**.

#### 2.5.1 Referral, inclusion, and pre-assessment

Participants were adolescents and young adults aged 15–21 years who were referred from outpatient clinics to the specialized transition clinic. Individuals with suspected or previously diagnosed NDDs were included. Both patients with established childhood diagnoses and those without a confirmed diagnosis but presenting with NDDs were evaluated.

Eligibility for referral to the transition clinic was based on two levels of criteria: core criteria, all of which were required, and additional clinical indicators, at least one of which had to be present. These criteria were determined based on clinical history and psychiatric examination and were intended to capture complex or atypical NDD presentations. The eligibility criteria are summarized in **Table 1**.

**Table 1:** Eligibility criteria for referral to the transition clinic.

Core criteria (all required)
• Age between 15 and 21 years, • Preliminary diagnosis of at least one neurodevelopmental disorder or diagnostic uncertainty,
Additional clinical indicators (at least one required)
• Atypical clinical course, • History of regression, • Parental consanguinity or recurrent pregnancy loss, • Known genetic diagnosis or unexplained sibling death, • Fluctuating course inconsistent with the clinical diagnosis, • Atypical side effects relative to medication dose, • Treatment resistance, • Multiple psychiatric diagnoses across different spectrum, • Functional decline not explained by psychiatric diagnosis, • Abnormal neurological examination findings, • Dysmorphic features, • Chronic systemic disease with solid organ involvement, • Marked microcephaly independent of familial traits, • History of epilepsy.

Before the interdisciplinary diagnostic evaluation, patients underwent a pre-assessment work-up. Psychiatry residents obtained detailed developmental history, psychiatric history, medical and surgical history, current and past medication use, and information regarding additional medical comorbidities. During this phase, clinical rating scales were completed by patients and/or their parents to assess the severity of comorbid psychiatric conditions, including anxiety and depressive symptoms. In addition, IQ testing and a comprehensive executive function/neurocognitive battery were administered by psychologists. Intellectual functioning was evaluated using the Wechsler Intelligence Scale for Children-Revised (WISC-R) (11) for adolescents and the Wechsler Adult Intelligence Scale-Revised (WAIS-R) (12) for young adults.

#### 2.5.2 Interdisciplinary diagnostic evaluation

Patients were evaluated jointly by a child and adolescent psychiatrist and an adult psychiatrist to ensure continuity of care and shared clinical decision-making. Psychiatric diagnoses were established according to DSM-5-TR criteria and using semi-structured clinical interviews.

The Schedule for Affective Disorders and Schizophrenia for School-Age Children, Present and Lifetime Version (K-SADS-PL) (13), was used for participants aged 15–18 years, whereas the Structured Clinical Interview for DSM-5-Disorders-Clinician Version (SCID-5/CV) was used for adult participants aged 18–21 years (14). Individuals evaluated for ASD underwent a detailed developmental history assessment involving both patients and their caregivers. ASD diagnoses were established according to DSM-5-TR criteria based on developmental history and comprehensive clinical evaluation by both the child and adolescent psychiatrist and the adult psychiatrist. Personality disorder diagnoses were established in accordance with DSM-5-TR criteria through developmental history and clinical evaluation.

All patients were subsequently evaluated by a consultant neurologist. When clinically indicated, additional investigations, including MRI, EEG, and laboratory tests, were performed. In cases where a genetic etiology was suspected based on clinical findings, patients were referred for further evaluation in medical genetics.

#### 2.5.3 Integrated treatment and follow-up

Following transition clinic evaluation, patients were followed either in the Adult Neurodevelopmental Disorders Clinic or the Adolescent Neurodevelopmental Disorders Clinic according to their age group. Pharmacological treatment planning, medical treatment follow-up, psychoeducation for patients and families, referrals for social skills interventions, and, when indicated, cognitive behavioral therapy and family interviews were integrated into the follow-up process.

### 2.6 Statistical analysis

Statistical analyses were performed using SPSS version 29. Descriptive statistics were calculated for sociodemographic and clinical variables. Continuous variables were presented as mean ± standard deviation, whereas categorical variables were reported as frequencies and percentages. Diagnostic distributions before and after transition clinic evaluation, neurological and genetic findings, and psychotropic medication use were analyzed descriptively. In addition, sex differences in newly identified diagnoses were examined using the chi-square test. Effect size was calculated using Cramer’s V.

## 3 Results

A total of 68 participants were included in the study. The mean age of the sample was 17.90 years (SD = 1.90; range = 15–21). The mean years of education was 11.37 (SD = 1.83; range = 6–15). The sample consisted of 31 females (45.6%) and 37 males (54.4%). The mean verbal IQ score was 103.93 (SD = 19.80; range = 64–140), and the mean performance IQ score was 91.73 (SD = 17.81; range = 61–138). The mean full-scale IQ score was 98.89 (SD = 18.19; range = 66–139).

**Figure 2:**
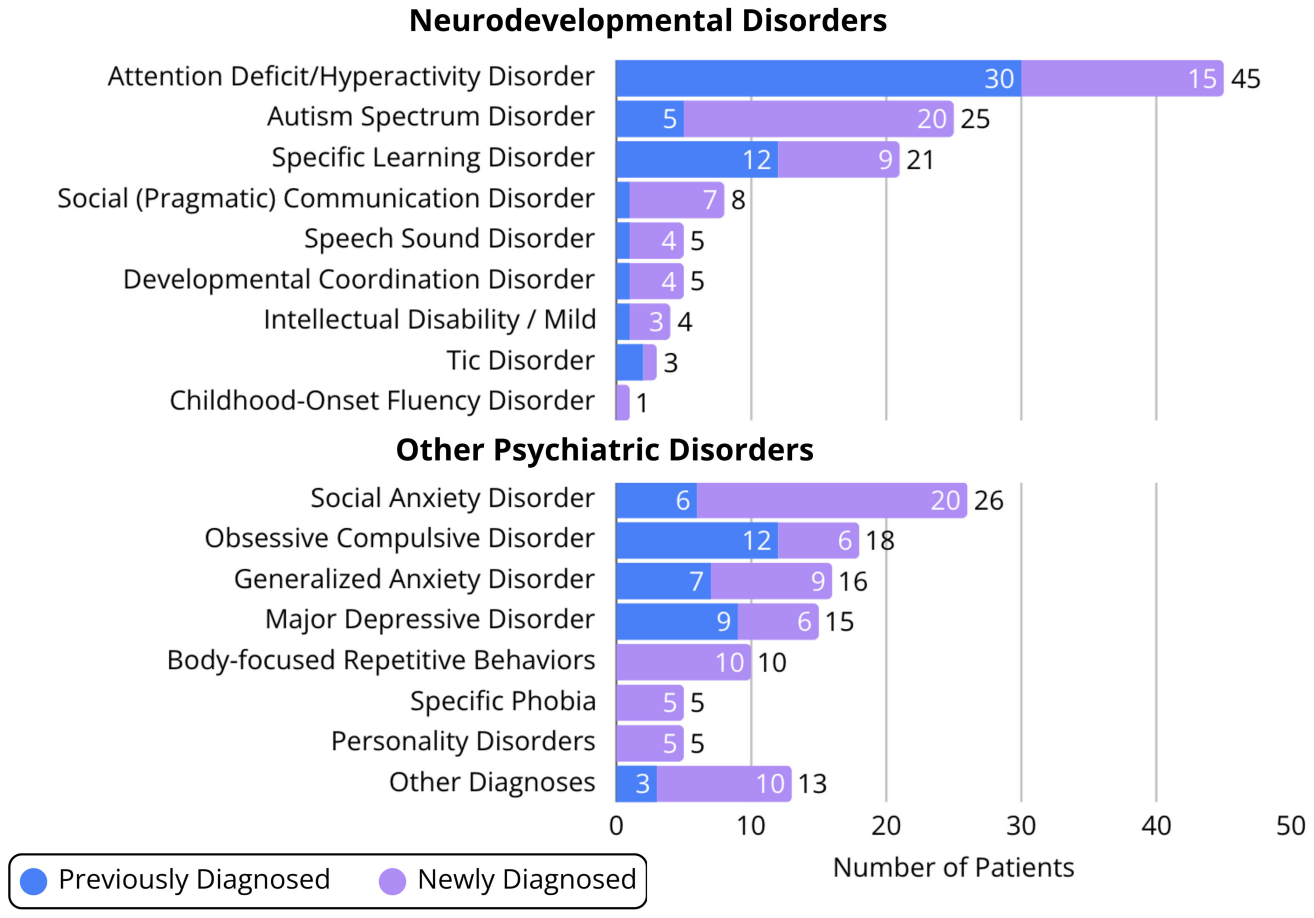
Distribution of psychiatric diagnoses in the transition clinic by diagnostic history. Newly diagnosed cases refer to individuals who received the diagnosis for the first time during the transition clinic assessment.

The median number of diagnoses increased from 1 (range: 0–4) before the transition clinic assessment to 3 (range: 1–11) after the assessment. The distribution of NDDs and other psychiatric diagnoses identified during transition clinic assessments is presented in **Figure 2**. ADHD was the most frequent NDD (n = 45), including 30 individuals with an established diagnosis and 15 cases identified during the transition assessment. ASD was observed in 25 patients, the majority of whom (n = 20) were identified at the transition clinic, while 5 had been previously recognized. Specific learning disorder was present in 21 individuals, including 12 with prior diagnoses and 9 detected during the evaluation process. A total of 26 diagnoses belonging to other NDD categories were identified, with 20 of these diagnoses being newly identified during the transition clinic assessment process. Among additional psychiatric disorders, anxiety disorders were the most frequent comorbidities (n = 47), with 34 cases identified during the transition assessment and 13 previously documented. Depressive disorders were observed in 15 patients, including 6 newly identified during the transition process. Body-focused repetitive behaviors (BFRBs) were identified in 10 individuals, all during the transition assessment. Personality disorders were identified in 5 participants, all of whom received their diagnoses during the transition evaluation (3 avoidant, 2 borderline). Other psychiatric diagnoses were observed in 13 individuals, including 10 identified during transition and 3 previously documented. A significant sex difference was observed for newly identified ASD diagnoses. Among participants without a previous ASD diagnosis, ASD was newly identified more frequently in males than in females during the transition clinic assessment (40.5% vs. 16.7%; χ²=4.51, p=.034, Cramer’s V = .259). No significant sex-related differences were found for other diagnoses.

Neuroimaging and electrophysiological evaluations were conducted in a subset of participants. MRI was performed in 30 individuals, of whom 8 showed structural neuroimaging findings. EEG was conducted in 28 individuals, with pathological findings detected in 1 case. Genetic testing (whole exome sequencing and chromosomal microarray analysis) was performed in 3 individuals, with one case showing a pathological finding.

**Figure 3:**
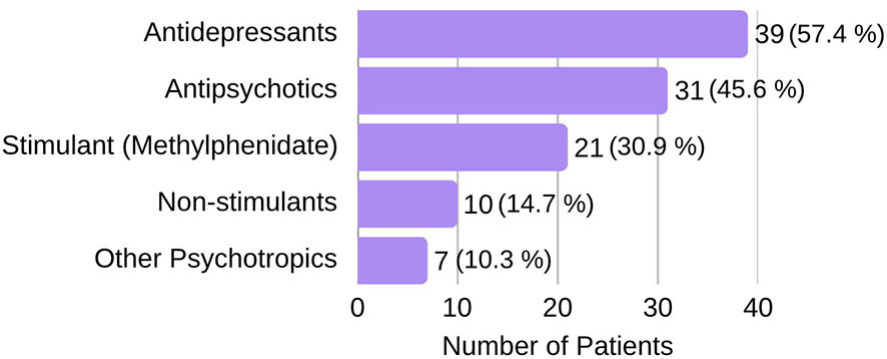
Distribution of pharmacotherapy in the transition clinic.

The distribution of psychotropic medication use among participants is presented in **Figure 3**. Antidepressants were the most commonly prescribed medications (57.4%), followed by antipsychotics (45.6%) and stimulants (30.9%). Non-stimulants (14.7%) and other psychotropic agents (10.3%) were used.

## 4 Discussion

The present study describes a structured, interdisciplinary transition clinic model for individuals with NDDs and presents preliminary findings from its implementation. The results indicate that a substantial proportion of both NDDs and other psychiatric diagnoses were newly identified during the transition clinic assessment, highlighting the clinical complexity of this population. The median number of diagnoses increased from 1 before transition assessment to 3 after evaluation. Especially, ASD, social anxiety disorder, social communication disorder, and BFRBs were among the most frequently newly identified diagnoses during the transition clinic assessment, suggesting that important diagnostic features may remain unrecognized during earlier phases of care. These findings support the need for comprehensive, interdisciplinary evaluation during the transition from child and adolescent to adult mental health services.

In our sample, among the 25 individuals diagnosed with ASD, 20 (80%) received their diagnosis during the transition clinic process. Considering that the typical age of ASD diagnosis ranges between 3 and 6 years—despite cross-national variability—and that early diagnosis is associated with better global functioning (15), the delayed age at diagnosis observed in our sample may have important adverse implications. When exploring the reasons underlying this delay, the absence of co-occurring intellectual disability in our sample may represent a key factor. Indeed, delayed diagnosis is more commonly observed in individuals with milder presentations and without language delay (16), as these individuals often exhibit more effective camouflaging abilities (17). Camouflaging behaviors may reduce the visibility of autistic characteristics by enabling individuals to mask autistic behaviors and compensate for social difficulties, potentially delaying clinical recognition despite the persistence of underlying impairments (18). Future studies may further examine the role of camouflaging behaviors during the transition period and their potential contribution to delayed recognition and diagnosis of ASD among transition-aged individuals. In addition, 12 out of the 25 individuals with ASD (48%) in our sample were also diagnosed with ADHD. This comorbidity may further contribute to diagnostic delay, as ADHD symptoms can obscure or overshadow core ASD features (15). Taken together, these findings suggest that increasing the number of transition clinics may be essential to prevent individuals from being “lost generation” (19) and to facilitate timely and accurate diagnosis. It should also be noted that our clinic primarily serves high-functioning individuals with diagnostically complex presentations in a tertiary-care setting, which may have contributed to the high rate of newly identified ASD diagnoses. Future multicenter studies involving a broader spectrum of NDDs are warranted to determine the extent to which these findings can be generalized.

Another important finding of our study was that ADHD was the most frequent NDD (66.2%), and 15 of 45 individuals received their diagnosis during the transition clinic assessment. The high prevalence of ADHD is consistent with the existing literature (20), the presence of cases diagnosed after the age of 15 is noteworthy. One possible explanation for this delay may be related to findings from a recent study indicating that, the predominantly inattentive presentation and greater maladaptive daydreaming severity are associated with delayed ADHD diagnosis (21). The assessment of maladaptive daydreaming within transition clinics may be both necessary and clinically relevant. In addition, limited training in NDDs during psychiatric training (10), together with resistance to ADHD diagnosis in some settings and stigma associated with seeking a diagnosis, may contribute to delayed recognition (22). From this perspective, transition clinics may play a critical role in mitigating clinician-related factors contributing to delayed or missed ADHD diagnoses, while also enhancing the training of psychiatric residents.

Other important findings of our study include that 87.5% of social (pragmatic) communication disorder diagnoses, 76.9% of social anxiety disorder diagnoses, and all cases of avoidant personality disorder were identified during the transition clinic process. We interpret this pattern in the context of adolescence, a developmental period characterized by increasing social demands and greater importance of social functioning. Especially late adolescence is characterized by the growing importance of peer and romantic relationships, which may place greater social demands on individuals and influence functioning (23). It has been reported that social (pragmatic) communication disorder and social anxiety disorder—particularly in milder forms—may remain unrecognized until adolescence or emerging adulthood, while avoidant personality disorder typically becomes more apparent in young adulthood (1). In this context, a comprehensive assessment of social skills, as well as the individual’s social interaction repertoire and reserve, appears to be particularly important within transition clinic evaluations.

Another notable finding was that both individuals diagnosed with borderline personality disorder received their diagnoses in our transition clinic. BFRBs meeting DSM-5-TR criteria were identified in 10 individuals, all during the transition assessment. Borderline personality disorder demonstrates a high comorbidity rate with ADHD (approximately 34%) (24) and, given its early onset along with underlying neurobiological, neurocognitive, and gene–environment mechanisms, has been suggested to fall within the NDD spectrum (25). The evaluation of borderline personality disorder within transition clinics appears warranted. With respect to BFRBs, a prevalence of 14.7% was observed in our sample, which is comparable to previously reported rates of approximately 13.7% in similar age groups (26). BFRBs are often perceived as shameful symptoms and are therefore frequently concealed by individuals (27). In this context, screening for personality features/disorders as well as BFRBs appears particularly important in adolescent and young adult populations within transition clinic settings.

Transition clinics may provide opportunities for resident education through exposure to newly identified and clinically complex cases. Limited awareness and knowledge among psychiatrists may contribute to delays in recognition and diagnosis. Previous literature has highlighted that many psychiatrists receive limited formal training regarding NDDs, which may contribute to reduced confidence in the recognition of associated psychiatric comorbidities (10). While participation in our transition clinic may have enhanced residents’ awareness and knowledge regarding NDDs, these educational effects were not formally evaluated through structured educational outcomes or resident feedback. Future studies incorporating standardized educational assessments and resident feedback may help clarify the educational value of transition clinics. In addition, diagnostic overshadowing is an important concept in NDDs and refers to the misattribution of clinically significant psychiatric symptoms solely to the underlying NDD, potentially leading to underrecognition of comorbid disorders. To reduce this risk, comprehensive, family-centered, and multidisciplinary approaches have been recommended in the assessment of individuals with NDDs (28). Similarly, NICE guidelines emphasize the importance of specialist multidisciplinary teams in the assessment and management of NDDs (29). In this regard, our transition clinic model appears to be consistent with these recommendations.

Our transition clinic incorporated neurological and genetic assessments as part of the interdisciplinary evaluation process. Several structural neuroimaging findings were identified during neurological evaluations; however, most appeared incidental or non-specific, and no consistent neuroimaging pattern emerged across participants. Identified findings included concha bullosa of the left middle turbinate, Ecchordosis physaliphora, retention cysts in the maxillary sinus, Rathke’s cleft cyst, intracranial hypertension, and nonspecific hyperintense lesions. Among 28 patients who underwent EEG, one demonstrated abnormal activity consistent with focal epileptiform dysfunction originating from the bilateral frontal and left temporo-occipital regions. In addition, a genetic anomaly was identified in one of the three individuals who underwent genetic testing. Although these findings provide preliminary information regarding neurological and genetic characteristics in transition-aged individuals with NDDs, their clinical significance remains uncertain. Given the relatively small sample size and the predominance of high-functioning individuals without co-occurring intellectual disability, further studies involving larger samples and individuals with a broader range of clinical and cognitive characteristics are needed to clarify the interpretation, specificity, and clinical relevance of these findings. Nevertheless, the inclusion of neurological and genetic evaluations may contribute to a more comprehensive understanding of complex NDD presentations within interdisciplinary transition services.

## 5 Limitations and future directions

First, the relatively small sample size and single-center design may limit the generalizability of the findings. In addition, the absence of a control or comparison group should be considered when interpreting the results. In this regard, international collaborations and the development of consensus-based frameworks may help strengthen the evidence base. Second, our sample predominantly consisted of individuals without comorbid intellectual disability and with relatively higher levels of functioning who were evaluated in a specialized tertiary-care transition clinic. Therefore, the findings may not be fully generalizable to all adolescents and young adults with NDDs, and the possibility of referral bias should be considered when interpreting the results. Future studies involving larger samples and individuals with varying levels of intellectual functioning would enhance the generalizability of the findings. Another limitation of the present study is the use of WAIS-R and WISC-R for cognitive assessment. Although newer versions of these instruments are available and recommended internationally, cognitive evaluations were based on the assessment tools routinely accessible within our clinical setting during the study period. Future studies may benefit from the use of more recent versions of standardized cognitive assessment instruments.

In addition, the potential educational benefits of this transition clinic model for psychiatry residents were not formally evaluated in the present study and may represent an important area for future research. Finally, the number of participants who underwent MRI, EEG, and genetic evaluation was relatively small, limiting the interpretation and clinical significance of these findings. Therefore, further studies with larger samples are needed to clarify the relevance of neurological and genetic findings in transition-aged individuals with NDDs.

## 6 Conclusion

An interdisciplinary transition clinic offers a novel framework for the comprehensive evaluation of individuals aged 15–21 years, enabling a multidimensional clinical perspective. The integration of neurology and, when indicated, genetic evaluation may also facilitate the identification of potential endophenotypes and help clarify which patients may benefit from specific investigations and at what stage. Overall, our findings may underscore the clinical and potential educational value of interdisciplinary transition clinics in improving the recognition and assessment of NDDs.

## Data Availability

The raw data supporting the conclusions of this article will be made available by the authors, without undue reservation.
